# Acquired factor XII deficiency following transanal excision of rectal lesion by transanal minimally invasive surgery (TAMIS): a case report and literature review

**DOI:** 10.1186/s12957-018-1410-x

**Published:** 2018-06-19

**Authors:** Maria Rita Cozzi, Andrea Lauretta, Roberto Vettori, Agostino Steffan

**Affiliations:** 1 Department of Immunopathology and Cancer Biomarkers, CRO National Cancer Institute, I.R.C.C.S, via Gallini, 2, 33080 Aviano, Italy; 2 Department of Surgical Oncology, CRO National Cancer Institute, I.R.C.C.S, via Gallini, 2, 33080 Aviano, Italy

**Keywords:** Transanal endoscopic surgery, Transanal minimally invasive surgery (TAMIS), Acquired factor XII deficiency

## Abstract

**Background:**

Local excision (LE) is currently one of the most effective methods used in cases of large benign polyps, not suitable for endoscopic treatment, or early-stage neoplasms. LE is also alternative to anterior rectal resection in selected patients suffering from major comorbidities and limits for major abdominal procedure. Furthermore, LE results in less pain, reduced impact on bowel function, shorter duration of hospital stay, and lower rates of morbidity, mortality and stoma creation. In particular, early data on transanal minimally invasive surgery (TAMIS) are promising, but they come from single centre case series related to small groups of patients and more data are needed to draw a final conclusion on the safety of this novel approach for transanal resection.

**Case presentation:**

A 62-year-old woman, following a positive faecal occult blood test and with unremarkable medical history, was admitted to hospital for excision of a large flat neoplastic lesion. Endoscopic biopsy demonstrated a tubular adenoma with high-grade dysplasia and was decided to proceed with surgical excision by TAMIS. After surgery, short-term outcomes revealed prolonged activated partial thromboplastin time, undetectable factor XII activity, fever, and partial dehiscence of rectal wall defect suture. Cross-mixing studies of patient plasma show no correction in either the immediate or incubated activated partial thromboplastin time, indicating the presence of an acquired factor XII inhibitor. Activated partial thromboplastin time and factor XII improved in the following weeks without any specific therapy in addition to antibiotic therapy.

**Conclusion:**

This is the first report in which acquired inhibitor of coagulation factor XII is associated with a specific surgical procedure. This case has shown how trans-anal excision of rectal lesions, even when performed by minimally invasive means such as in case of TAMIS, is not free of complications. We consider the acute infection, resulting from early dehiscence of the suture, the trigger in an abnormal immune response, and inhibitor development.

## Background

Over recent years, colorectal cancers are frequently diagnosed at early stages, mostly in countries where is operative cancer screening programmes. For large benign polyps and early stage rectal cancer, local excision (LE) represents a valuable alternative to radical resection and more recently has also been offered to patients following neoadjuvant chemoradiation [1].

In these cases, LE is ideal for the better short-term results (mortality and morbidity within 30 days of surgery), is associated with less pain, less impact on bowel function, and stoma creation. Different techniques are used for LE including conventional local excision, transanal endoscopic microsurgery (TEM), and transanal minimally invasive surgery (TAMIS).

The first series of patients who underwent a TAMIS surgery was published in 2010 [2], and there has been a steady increase in the use of TAMIS over recent years.

However, despite its increasing adoption, there is little evidence in the literature on early complication and the sample size in published series is small; hence, surgical outcomes are subject to variation. We report the first case, to our knowledge, of acquired factor XII (FXII) deficiency due to a specific inhibitor development following transanal excision of rectal tubular adenoma by TAMIS.

## Case presentation

**Fig. 1:**
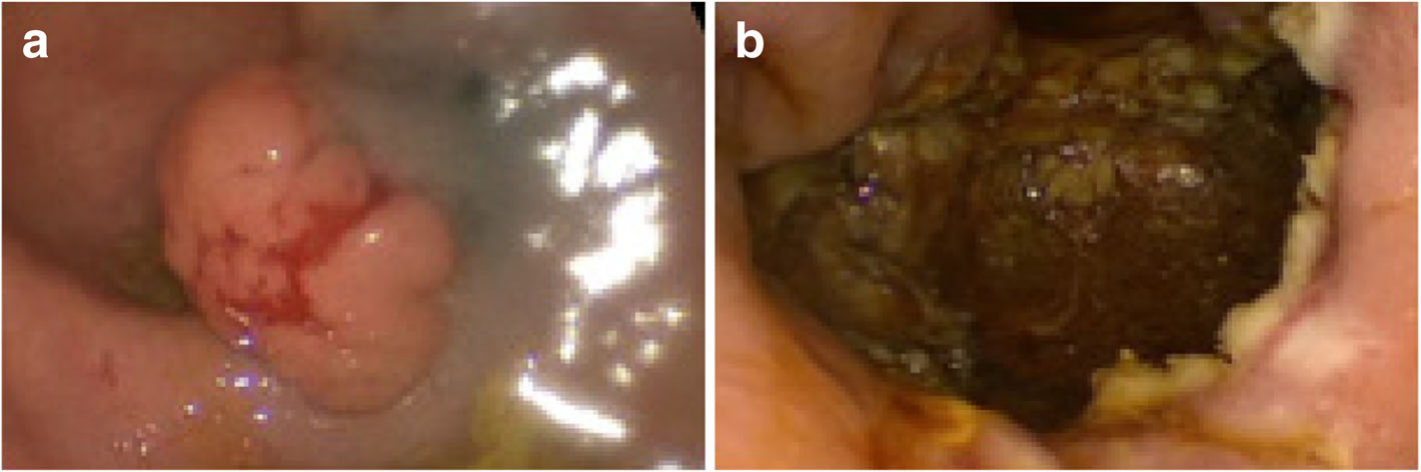
**a** Intraoperative view of the lesion. **b** Rectal defect following removal of the lesion

The present case involves a 62-year-old woman admitted to surgical oncology unit for a planned transanal excision of a large polyp of the mid rectum. Following a positive faecal occult blood test, colonoscopy detected the presence of a large flat neoplastic lesion, 50 mm in maximum diameter, tending to grow laterally and involving one-third of the rectal lumen (Fig. 1a). The lesion was located in the mid rectum, 8 cm from the anal verge and, based on its detailed endoscopic appearance during chromoendoscopy, was labelled as a lateral spreading tumour granular type (LTS-G). The endoscopic biopsy demonstrated a tubular adenoma with high-grade dysplasia. In view of the size of the lesion, endoscopic mucosal resection was considered unfeasible and it was decided to proceed with surgical excision transanally by TAMIS. The day before surgery, patient had standard mechanical bowel preparation and at the time of anaesthetic induction received preoperative antibiotics (Cefazolin 2 g and Metronidazole 500 mg). The procedure was performed under general anaesthesia and the single incision laparoscopic surgery port (SILS™ Port, Covidien) was adopted and traditional laparoscopic instruments were used. The surgery lasted 2 h with no intraoperative complications. The rectal wall defect was washed with a povidone-iodine solution (Fig. 1b) and then closed by a running suture performed with a barbed suture (Covidien V-Loc™).

Patient had unremarkable past medical history and on admission routine laboratory profile was in normal range: WBC, 6.34 × 10^3^/μL (reference value, 4–10 × 10^3^/μL); platelets, 231 × 10^3^/μL (reference value, 150–400 × 10^3^/μL); prothrombin time (PT), 11.4 s (reference value, 10.0–13.4 s); activated partial thromboplastin time (APTT), 34 s (reference value, 22.0–43.0 s); fibrinogen, 301 mg/dL (reference value, 160–450 mg/dL). Following surgery, the patient was allowed to be mobilised and to have a regular diet with no restriction, and standard prophylaxis for venous thrombosis was started with low molecular weight heparin (LMWH).

On day 3, the patient developed a spike in temperature without any suspicious clinical evidences. She was passing flatus associated with the mucous discharge, the abdomen was soft and not tender, and digital rectal examination did not show any lump or collection. Laboratory data disclosed both a deranged coagulation profile with marked APTT prolongation (126 s), PT 12.5 s, fibrinogen 897 mg/dL, raised white blood cells count (WBC 21.00 × 10^3^/μL) and procalcitonin 0.52 ng/mL (reference value, < 0.5 ng/mL). Cross-mixing studies of patient plasma and normal pooled plasma (25, 50, and 75%) insufficiently corrected the APTT (99, 71, and 56 s, respectively) after 2 h of incubation at 37 °C. Lupus anticoagulant assay was negative by dilute Russel viper venom test (DRVVT). FVIII, FXI, and FIX were in normal range whereas coagulant activity of FXII was < 1%, tested using one-stage APTT-based clotting assay.

**Fig. 2:**
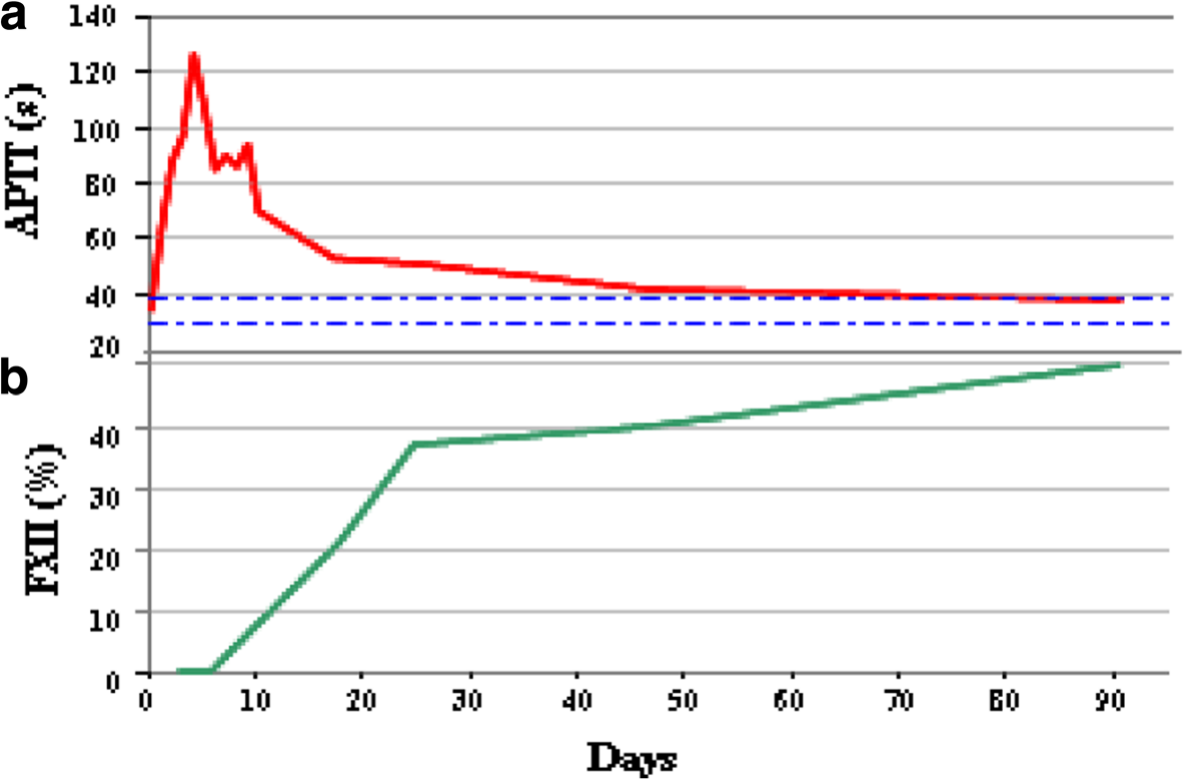
Time course of APTT (s) and FXII activity (%) during follow-up after surgery

Considering the spike in temperature and laboratory data, a computed tomography (CT) of abdomen and pelvis was performed to rule out any collection and source of infection. The CT scan did not show any pelvic abscess, but there was evidence of perirectal fat suffusion related to the recent procedure. A rigid proctoscopy was performed showing the evidence of the partial dehiscence of rectal wall defect suture; no other abnormalities were noticed. Antibiotic therapy was started with intravenous ciprofloxacin and metronidazole (500 mg) three times a day. From a therapeutic point of view, there is a general consensus that patients with an FXII inhibitor do not need any correction of the APTT and so our patient did not receive any therapy in addition to antibiotics. LMWH standard prophylaxis for venous thrombosis, initially suspended, was resumed. In the next 7 days, the patient had no more fever and laboratory data improved while APTT was still prolonged (70 s). She was discharged home with no further intervention. The histopathological report demonstrated a tubular adenoma with low- and high-grade dysplasia with free excision margins. 45 days later endoscopy showed a complete mucosal healing, APTT and FXII activity were back to the normal value (38 s and 50%, respectively). Time course of APTT and FXII activity during follow-up after surgery is pictured in Fig. 2.

Blood and tissue samples used were prepared and stored by CRO-Biobank (CRO National Cancer Institute, Aviano, Italy).

## Discussion

TAMIS consent accurate transanal excision of rectal lesions through of a single incision access-port and traditional laparoscopic instruments [2]. A recent systematic review including 390 cases showed a higher rate of negative microscopic margins, reduced rate of specimen fragmentation, and reduced recurrence compared to conventional transanal excision [3]. TAMIS also seems to improve anorectal function based on baseline questionnaires and showing a quality of life equal to the general population [4]. Furthermore, this surgical approach has a low rate of complications and the most common are not serious; other uncommon but harmful complications have been described as peritoneal entry or rectovaginal fistulas with the need of stoma formation [5]. A recent study reported overall 30-day morbidity of 16% including small suture line perforation, postoperative rectal bleeds, and urinary tract infection [6].

In the postoperative period following TAMIS fever is a frequent event and in most patients, as in ours, it is transient and not followed by the development of septic complications. The cause of early postoperative fever is unknown and currently, the hypothesis is that it is related to transient bacterial translocation after the procedure [7].

Here, we discuss a case of a patient who presented prolonged APTT associated with undetectable FXII activity and fever. The patient’s plasma cross-mixing studies show no correction of APTT and demonstrate the presence of an acquired FXII inhibitor. To our knowledge, this is the first report of a particularly rare acquired coagulation factor inhibitor associated with transanal surgical excision, including all available transanal approaches.

Acquired inhibitors of coagulation prevent the activity or increase the clearance of single clotting factor, and they are predominantly directed against FVIII, FIX, or VWF developing the acquired haemophilia or the von Willebrand’s syndrome. The incidence of acquired defect of other clotting factors like FII, FV, FVII, FX, FXI, FXII, and FXIII is much less known since only sporadic cases have been described. Autoimmune disease, solid tumours, lymphoproliferative, and inflammatory bowel disease are usually associated with the presence of such inhibitors [8]. A specific inhibitor of factor XII have been described, most frequently in association with the liver or gastrointestinal diseases and remission of the underlined diseases usually suppressed the inhibitor production [9, 10]. Plasma protein FXII, known as Hageman factor, in its active form (FXIIa) starts the intrinsic pathway of blood coagulation and with high molecular weight kininogen (HK), prekallikrein (PK), and complement factor C1q rise inflammatory responses involving blood coagulation, fibrinolysis, and inflammatory kinins [11, 12]. Individuals with FXII defect are characteristic because they have prolonged APTT, but FXII do not appear to require in-vivo for normal haemostasis. These subjects a have regular haemostatic capacity and the absence of bleeding tendency has led to the assumption that the in vivo fibrin formation is initiated predominantly through the extrinsic way of coagulation [13]. Therefore, FXII plays an important role in the clot formation in vitro as evidenced by the prolonged APTT, while its role in vivo is not proven.

Based on these backgrounds, even though the activated partial thromboplastine time (APTT) was abnormally prolonged, it was not corrected.

In this case, we postulated that the development of inhibitor was closely related to acute infection after local excision of the rectal tumour. One explanation is that rectal wall defect, occurring following suture dehiscence, acts as an open door for bacterial translocation developing an abnormal immune response to fight off an invading bacterium. Bacterial contamination of rectal fat leads to exaggerated immune response and specific coagulation inhibitor development. However, the relationship between coagulation FXII inhibitor and infection is not clear yet. Then we consider the infection as “primum movens” in the inhibitor development after the rectal wall violation.

The closure of the rectal wall defect does not seem to be a mandatory step of the procedure since no closure does not raise postoperative complications rate [14]. Furthermore, the closure is also technically demanding and time-consuming, leading the surgeon to often give up to this part of the procedure. While the closure of rectal wall defect is relatively easy during traditional local excision, a laparoscopic suture is most of the time challenging even for experienced surgeons and this becomes even more difficult during TAMIS where the space for triangulation of instruments is reduced significantly. In our case, the defect was closed but an early dehiscence of the suture occurred allowing thus bacterial contamination of the perirectal fat. Even though two recent papers [14, 15] highlighted no significant advantages with rectal wall defect closure, we suggest to close, when possible, the surgical wound in the rectum to avoid septic complications.

## Conclusion

TAMIS, is a new method to perform local excision safely. It offers improved outcomes in term of morbidity and functional outcomes for treatment of colorectal cancer. The rectal wall defect closure following excision is a well-known challenge even for expert surgeons due to the small surgical room and limited instruments movements specific of this procedure. However, a rectal wall defect left open remains a source of potential complications. In conclusion, when feasible, closure of the defect is not only appropriate but also recommended.

Finally, this case highlights the importance of laboratory differential analysis to identify any acquired coagulation factor inhibitors with a careful and systematic approach that excludes other possible causes of prolonged screening tests. An accurate diagnosis provides the best treatment in order to prevent bleeding symptoms and unwarranted transfusion after surgery; furthermore, it may provide a tailor-made approach to prevent thromboembolic risk for each patient.

## Abbreviation

APTT: Activated partial thromboplastine time
FXII: Factor XII
LE: Local excision
TAMIS: Transanal minimally invasive surgery
